# Intervertebral Disc Diseases PART 2: A Review of the Current Diagnostic and Treatment Strategies for Intervertebral Disc Disease

**DOI:** 10.3390/ijms21062135

**Published:** 2020-03-20

**Authors:** Pang Hung Wu, Hyeun Sung Kim, Il-Tae Jang

**Affiliations:** 1 Spine Surgery, Nanoori Gangnam Hospital, Seoul 06048, Korea; wupanghung@gmail.com (P.H.W.); nanooriresearch@gmail.com (I.-T.J.); 2 Orthopaedic Surgery, Jurong Health Campus, National University Health Systems, Singapore 119228, Singapore

**Keywords:** intervertebral disc, degenerative disc disease, spinal molecular therapy, stem cells, spinal fusion, spinal decompression, spinal endoscopy, spine pain management, spinal injection, gene therapy

## Abstract

With an aging population, there is a proportional increase in the prevalence of intervertebral disc diseases. Intervertebral disc diseases are the leading cause of lower back pain and disability. With a high prevalence of asymptomatic intervertebral disc diseases, there is a need for accurate diagnosis, which is key to management. A thorough understanding of the pathophysiology and clinical manifestation aids in understanding the natural history of these conditions. Recent developments in radiological and biomarker investigations have potential to provide noninvasive alternatives to the gold standard, invasive discogram. There is a large volume of literature on the management of intervertebral disc diseases, which we categorized into five headings: (a) Relief of pain by conservative management, (b) restorative treatment by molecular therapy, (c) reconstructive treatment by percutaneous intervertebral disc techniques, (d) relieving compression and replacement surgery, and (e) rigid fusion surgery. This review article aims to provide an overview on various current diagnostic and treatment options and discuss the interplay between each arms of these scientific and treatment advancements, hence providing an outlook of their potential future developments and collaborations in the management of intervertebral disc diseases.

## 1. Introduction

**Figure 1:**
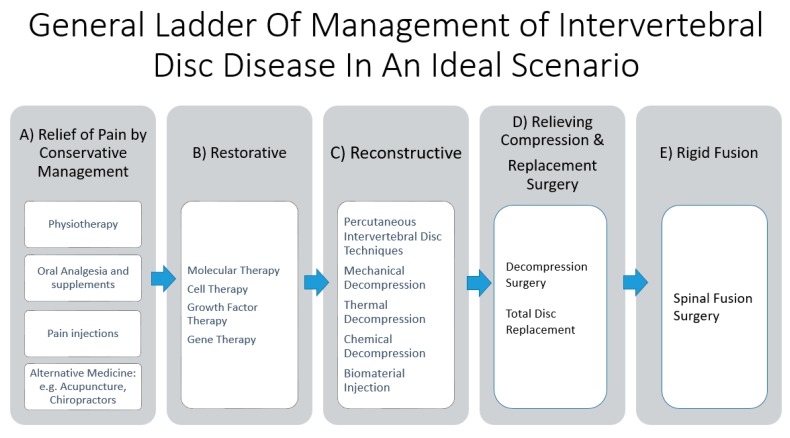
General ladder of management of intervertebral disc disease in an ideal scenario. (**A**) Relief of pain by conservative management comprises of physiotherapy, oral analgesia, and nutrition supplements supplemented with or without pain injections. Alternative medicine, such as acupuncture and chiropractor practice, has been described but it is beyond the scope of this review. (**B**) Restorative therapy involves molecular therapy, such as gene, growth factor, and cell therapy, which aim to restore the anabolic function and decrease the catabolic function of the disc in an attempt to repair the disc damage. (**C**) Reconstructive therapy involves reshaping the disc through decompression, such as mechanical, thermal, and chemical decompression, and biomaterial injection, such as polyvinyl alcohol cryogel, which has biomechanical properties similar to an intervertebral disc. (**D**) Relief surgery, such as decompression surgery, relieves compression of the neural elements from the associated pathological thickening of the ligamentum flavum or bulging disc from degenerative disc disease. Replacement surgery, such as total disc replacement, removes the affected diseased disc segment and replacing it with an artificial disc, hence preserving the motion of the lumbar spinal segment. (**E**) Rigid fusion surgery is the most invasive and definitive management, where the motion of the spinal segment is sacrificed, with the intervertebral disc removed and replaced with bone grafts to facilitate fusion of the lumbar segment. It is of note that many of the restorative and reconstructive therapies are under experimental and clinical trial stages; widespread practice on the general population is not recommended unless more evidence is gathered. Hence, only in the “ideal” situation when all the treatment options are available, can we fully utilize this ladder of management.

Lower back pain remains the most important musculoskeletal condition affecting the quality of life over the past few decades [1]. Up to 84% of the population have back pain at some point in their lives [2]. Some of the most common causes of lower back pain are intervertebral disc (IVD) diseases and their associated pathologies. IVD can affect both the young and old population [3,4]. Treatment strategies need to consider age of presentation, comorbidities, severity of IVD, neural elements compression and stability of the spinal column. It is a complex algorithm of management, with confounding factors contributing to success in the management of symptoms. It is in the authors’ opinion that the general stepwise invasiveness ladder of management in the treatment of IVD diseases is the relief of pain by conservative management, restorative, reconstructive, replacement, and finally rigid fusion (Figure 1). There is robust interest in clinical and basic science research of different treatment strategies of IVD management. Many of the restorative and reconstructive management strategies are still at the early stages of laboratory experimental and animal trials, with clinical efficacy yet to be proven. That is despite the fact that the prevalence of disc degeneration was found in at least one lumbar level in 35% of subjects between 20 and 39 years of age and all subjects 60 to 80 years of age showed disc degeneration in a study done by Boden et al. [5]. Degenerative disc disease (DDD) and prolapsed intervertebral disc (PID) are the two commonest forms of IVD diseases. They have a close cause and effect relationship as a prolapsed intervertebral disc is a risk factor of degenerative disc disease while advanced degenerative disc often presents with disc prolapse with annular fissure due to degeneration leading to a fragmented disc being prolapsed into the spinal canal [6]. There are a number of studies and reviews on specific arms of diagnostic and treatment strategies in intervertebral disc diseases. However, there is a paucity of literature giving a broad summary of the interplay in these developments of the diagnostic and treatment strategies. This review’s goal is to provide a broad overview of the various issues in intervertebral disc diseases with the proposed treatment strategies and a categorization of the various developments in the management of intervertebral disc diseases.

## 2. Degenerative Disc Disease

Disc degeneration is an abnormal structural failure as a consequence of the cell-mediated response to multifactorial contributions, such as genetics, micro/macro trauma, accelerated age-related changes, inflammation, local nutritional deficiency, and vascular factors, leading to excess catabolic over anabolic responses. Degenerative disc disease is a painful degenerated disc. Early degeneration shows changes in the matrix of the nucleus pulposus and annulus fibrosus [7]. The exact tipping point leading to a shift from anabolic to catabolic activities is unclear, but studies showed there are proinflammatory changes with increased inflammatory cytokine and nociceptive cytokine production. Neurotization of sinuvertebral nerve and basivertebral nerve infiltration along with granulation tissue acts as a response to these cytokines, leading to painful sensation from degenerative disc disease [7,8,9,10,11,12].

## 3. Prolapsed Intervertebral Disc

The prolapsed intervertebral disc was elaborated in our part 1 series explaining the types of disc herniation [12]. Disc material that is prolapsed can be part of the nucleus pulposus, annulus fibrosus, end plates material, or a combination of the above. These prolapsed disc materials can cause a significant inflammatory response and nerve root irritation [13,14]. The physical narrowing of the spinal canal coupled with the inflammatory response leads to the symptoms of radicular pain and sciatica [15].

## 4. Clinical Findings

Degenerative disc disease patients typically present with mechanical lower back pain, which is worse on forward flexion and when carrying heavy load. The pain is relieved with rest and lying supine with calves supported on a pillow. Advanced degenerative disc disease can present with morphological changes in the spine, such as intervertebral disc bulge, disc herniation, facet hypertrophy, and thickening of the ligamentum flavum, which in turn can lead to spinal stenosis and neural compression. The presentation of symptomatic spinal stenosis is neurogenic claudication where the patient presents with radicular pain, which is distance and time limited, and the pain is relieved by sitting or flexing the hip and knee at rest as these actions widen the spinal canal and relieve the compression on the neural elements. Disc herniation presents with radicular pain that radiates from the buttock in a dermatomal distribution down one or both legs. When the radicular pain presents on both legs, it is important to rule out a large disc herniation, which bilaterally affects the legs’ dermatomes. Such a large disc herniation may have effects on the continence of the urinary and bowel function if significant compression of the neural elements is present. The severity and extent of pain is not a direct correlation of the size of the disc and degree of neural compression but rather a combination of various factors, such as chronicity, size, and the type of materials herniated in the prolapsed intervertebral disc. It is possible that a small disc herniation pressing on dorsal root ganglion can lead to severe pain while a large chronic central disc herniation is asymptomatic [16,17,18]. Cauda equina and conus medullaris are an uncommon emergent presentations of a prolapsed intervertebral disc, which are associatedwith high canal compromised disc herniations. The symptoms are urinary and bowel incontinence, which are catastrophic presentations for these patients who require early surgical intervention [19,20].

## 5. Natural History: Degenerative Cascade

Kirkaldy-Willis et al. first described the degenerative cascade. The cascade starts with circumferential annular fissures propagating radially with the herniation of materials into the perineural space. This leads to a loss of disc height. The loss in disc height causes more stress on the facet joints and redundancy of the annulus, posterior longitudinal ligament, and ligamentum flavum, which leads to buckling into the spinal canal. Over time, the ligamentum flavum may thicken, leading to compression on the neural elements. The facet joint is a synovial joint, which reacts to excess stress on the joint from the decreased disc height, leading to progression with osteoarthritis processes, with a loss of joint space, osteophytes formation, and subchondral articular cartilage degeneration as a result. Loss of articular cartilage leads to articular capsule laxity and propagates into joint subluxation. The spine unit attempts to stabilize the IVD and facet joints by hypertrophic osteophytes formation around the facet joints and periarticular fibrosis, leading to stiffer facet joints. Despite the best efforts in containing the instability, the process continues to deteriorate with the overriding facet occupying and narrowing the foraminal space and spinal canal compression with osteophytes, syndesmophytes, buckling of the thickened ligamentum flavum, and protrusion of the disc [21].

## 6. Current Diagnostic Imaging

### 6.1. Role of Roentgenogram and Magnetic Resonance Imaging

**Figure 2:**
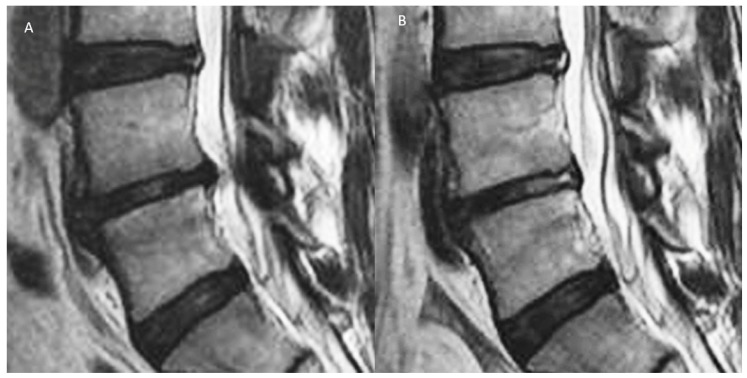
**A**: Degenerative disc disease with Pfirrmann grade III disc showing an inhomogeneous structure, and an unclear distinction of nucleus and annulus and type 1 Modic changes with intermediate signal intensity in the T2 image with slightly decreased disc height and disc bulge. **B**: After radiofrequency ablation of the disc, sinuvertebral nerve, and basivertebral nerve, there is shrinkage of the degenerative disc and there is an increase in the signal of Modic changes in the adjacent vertebra body. However, with the current imaging technique, there are limitations in quantifying the effects of these end plate changes objectively. Further development in this area of assessment would be beneficial to assess treatment effects on the end plate and disc in early DDD (figure reproduced with permission courtesy of Kim et al. [12]).

Plain roentgenogram (X-ray) is useful in the diagnosis of DDD. There are radiological signs of a decrease in disc height, associated syndesmophyte formation in the adjacent vertebral body, facet hypertrophy, and spondylolisthesis. The flexion and extension view aids in establishing the degree of instability in the spine. Magnetic resonance imaging (MRI) is the standard imaging modality in detecting IVD diseases. It has advantage of being radiation free and allows a multiplanar evaluation, with good soft tissue contrast giving more accurate interpretation of disc changes [22]. The common MRI classifications used by spine surgeons in the interpretation of degenerative disc disease and its related problems is the Pfirrmann classification for disc morphology [23] and Modic classification for adjacent vertebral body changes [24]. The Pfirrmann classification demonstrates the stages of changes in disc degeneration progression from one stage to the next, becoming more progressive in the destruction of disc architecture, as one moves from grade I to V. Generally, grades I–III are early disc degeneration and grades IV–V are advanced disc degeneration. Modic changes are a reflection of the active inflammation and fibrovascular replacement of the hematopoietic marrow. It appears hypointense on T1-weighted imaging and hyperintense on T2-weighted imaging, known as type 1 Modic changes. The fatty replacement of the end plate lesion appears with hyperintense T1 and isotense in T2 in type 2 Modic changes. Sclerotic end plate changes are hypointense in both T1 and T2 images in type 3 Modic changes. Modic changes can be transient or permanent changes in a chronic pathological process. They are closely correlated to lower back pain and occur more commonly in the lumbar spine. Figure 2 shows that the clinical importance of Pfirrmann and Modic classification is controversial, but they remain useful radiological parameters to indicate the severity of DDD [25,26,27].

### 6.2. Recent Developments in Diagnostic Tool Imaging

Recent developments in a more granular assessment of MRI and Computer Tomography scan may provide a glimpse of the future assessment of various stages of DDD and the treatment effects on the natural history of DDD. Kim et al. described that with the endoscopic radiofrequency ablation as a treatment for DDD-related basivertebral and sinuvertebral nerve pain, in both the morphology of the disc and adjacent end plate, Modic changes were observed (Figure 2). New radiological techniques in detecting more subtle changes in disc and related tissues can symbiotically allow better advancements in restorative and reconstructive techniques to provide radiological outcome assessment of these new interventions in DDD.

#### 6.2.1. Contouring of Modic Changes

The manual contouring of Modic changes in mid sagittal slices has good intra and inter-rater reliability as demonstrated by Wang et al. [28]. Semi-automated contouring is less labor intensive and enhances the reliability of indices of Modic size changes [29]. Such quantitative measurement rather than qualitative measurement may help improve the reliability of the assessment of Modic changes.

#### 6.2.2. Assessment of Bone Marrow Lesion Composition

One can objectively classify end plate bone marrow lesions based on the composition of the bone marrow rather than the size or structure of the lesion. MRI based on chemical shift encoding-based water–fat imaging enables the spatially resolved assessment of bone marrow fat at trabecular sites, with a heterogeneous red marrow distribution giving an indication of the bone marrow involvement and assessment of the bone marrow lesions. It also allows for better monitoring of the bone marrow response to treatment in DDD [30,31].

#### 6.2.3. Diffusion Tensor Imaging

Diffusion-weighted imaging (DWI) can provide valuable information regarding the microstructure of tissues by applying a motion probing gradient (MPG) in some directions to monitor the random movement of water molecules, which is restricted in tissues. Water molecules tend to move along the nerve fibers in neural tissue, and this is called anisotropic diffusion. Diffusion data can be used to analyze and visualize nerve roots, but it is still in the investigational stages [32]. Studies showed it is a useful tool in peripheral nerves [33] and peripheral nerve degeneration and regeneration [34]. Particularly, fractional anisotropy values are useful interpretations for axon-related and myelin-related injuries and recovery. The mean fractional anisotropy of entrapped symptomatic nerves was less than that seen on the intact side, demonstrating that the tractography shows abnormal findings for nerve roots in lumbar spinal degeneration and that FA decreases in symptomatic roots [35]. Recently, zoomed echo-planar diffusion tensor imaging for MR tractography was studied by Ream et al. and had gained improved qualitative and quantitative measures of image and tract fiber quality. These techniques can be explored in a careful assessment of neural adhesions and the relationship of nerve fibers with DDD, especially in the advanced stages of DDD.

#### 6.2.4. Multi-Detector Computed Tomography Scan

The progression of intervertebral disc (IVD) degeneration leads to disc rupture within IVD tissues. The location and appearance of areas of gaseous radiolucency in the IVD, known as vacuum phenomena (VPs), are considered to indirectly indicate the position and extent of IVD rupture. The disc height tends to be lower in discs that contain VPs, especially in the anterior AF area. The shape and distribution of intradiscal VPs wer significantly associated with the degree of disc degeneration and lumbar spinal stenosis, as graded by MRI. Discs with VPs extending from the NP into the anterior and/or posterior AF had a significantly higher proportion of advanced disc degeneration (Pfirrmann’s classification: grades IV and V) [36]. This is a potentially useful assessment tool in the future, especially in patients who are contraindicated to undergo an MRI scan in the assessment of DDD. More studies and classification on multi-detector computed tomography scan assessment of DDD are needed in the future.

## 7. Development of Biomarkers

Current assessment of DDD is mainly based on clinical and radiological assessment. Specific biomarkers for symptomatic DDD have the potential to monitor the response to treatment using various restorative and reconstructive techniques, which will synergize the development of early phase symptomatic DDD. Degenerative disc disease and prolapsed intervertebral disc conditions cause morbidity through both physical changes compressing neural elements and inflammation secondary to inflammatory mediators. Hence, studies on inflammatory biomarkers have gained significant scientific interest, with several studies showing that various biomarkers have correlations with degenerative disc disease and prolapsed intervertebral disc.

For disc herniation, Park et al. showed Interleukin( IL)-2, IL-6, and IL-8 were raised four-fold, and Tumor Necrosis Factor(TNF)-α raised two-fold when compared with controls [37]. Kraychete et al. showed TNF-α was raised six-fold and IL-6 raised four-fold higher than the control group [38]. Pedersen et al. showed IL-6 and IL-8 are higher in high pain compared to the low pain group and IL-6 decreased over time in the high pain group [39]. Other markers, such as(T-Helper) TH17 and IL-17 [40], IL-21, and IL-17 [41], have been shown to increase with a prolapsed intervertebral disc.

For disc degeneration, Goode et al. showed type II collagen and Cartilage Oligomeric Matrix Protein COMP are associated with disc space narrowing [42]. Grad et al. showed Chemokine (C-C motif) ligand CCL5 was 1.6 times higher in the degenerated group compared with non- degenerated controls; CXCL6 was 1.3 times higher in the degenerated group compared with the control [43]. Ye et al. showed IL-18 increased with worsening disc degeneration [44].

Overall, though inflammatory biomarkers are at the early phase of development, it is a promising field, which can be a game changer in deciding which patients are at risk of further progression of disc disease and which patients are going to be more symptomatic [45]. Clinical applications of inflammatory biomarkers can be applied to look for downward trending responses in biomarkers after restorative and reconstructive intervention in IVD diseases. More studies are required to look for a reliable response of biomarkers to the recovery of IVD after treatment.

## 8. Standard Confirmatory Tests

**Figure 3:**
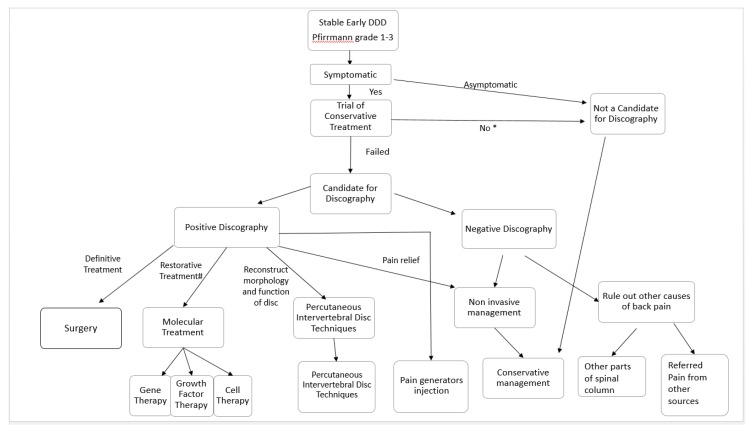
Decision flow chart in clinical practice for patients with stable early degenerative disc disease. Patients with early DDD manifested as Pfirmann grade 1–3 disc on MRI are assessed for symptoms. Asymptomatic patients and symptomatic patients who have not tried conservative management (*) should undergo a trial of conservative management first. Patients with persistent discogenic pain who failed conservative treatments can be considered for discography. A negative discography makes disc-related symptoms less likely. One should consider ruling out other causes of back pain related to the spinal column, such as facet joint pain, tumor, infection, and fracture, etc., or referred pain from other visceral organs, or musculoskeletal back pain. A positive discography is suggestive of disc-related symptoms. This group of patients are consulted for options of invasive or noninvasive treatment strategies. A noninvasive conservative management option for pain relief consists of physiotherapy, oral medications, exercises, and alternative medicine. Invasive options include pain relief by pain generator injections. Injection of steroid and local anesthesia can be applied to pain generators, such as facet block to the facet joint, epidural steroid injection to the epidural space, and peri radicular injections for neuropathy or percutaneous epidural neurolysis when adhesion between the dura and intervertebral disc diseases is observed. Reconstruction of disc morphology, with percutaneous intervertebral disc techniques, such as mechanical, chemical, and thermal decompression and biomaterial injection, is another option. Many of these therapies are still in experimental stages. Restorative treatment, such as molecular treatment, such as gene, growth factor, and cell therapy, is another option, but most of these therapies are still at the clinical trial stages. Surgery is often the last definitive treatment option if other conservative treatment strategies fail.

The confirmatory tests described in the literature are immobilization in a lumbosacral orthosis, provocative discography, and trial immobilization by temporary external transpedicular fixation [46]. Comparing the three tests, a trial of immobilization by orthosis is the least invasive; however, it is not able to determine the correct level of DDD in a patient with multiple levels of DDD. External Transpedicular fixation is cumbersome and invasive, hence it is not preferred by most patients and surgeons. Hence, provocative discography is the most popular option in differentiating the diagnosis of discogenic back pain from non-specific back pain in patients who have degenerative disc disease [47,48] (Figure 3).

## 9. Current Treatment Strategies for Degenerative Disc Disease

**Figure 4:**
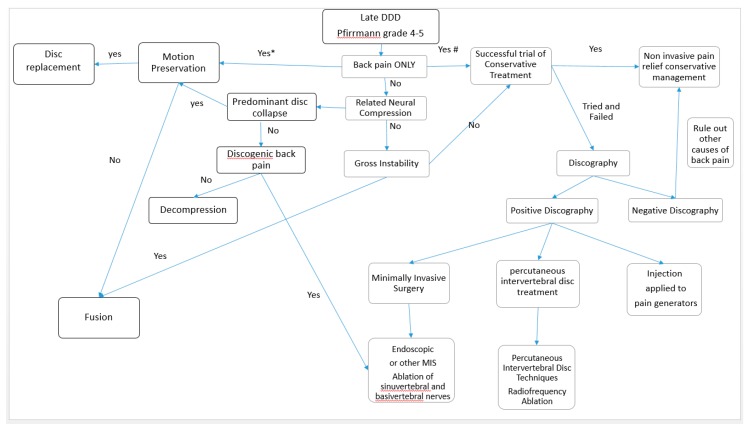
Decision flow chart in clinical practice for patients with late degenerative disc disease, Pfirrmann grade 4–5. We assess for any related pure axial back pain, neural compression, and instability. If there is no neural compression or instability and only pure axial back pain, the patient should be offered a trial of conservative noninvasive treatment as a first line of treatment (#). It is controversial regarding total disc replacement and fusion surgery as a first line of definitive treatment for back pain. Both are invasive and irreversible procedures; hence, they should be considered as a last option in pure axial back pain even in advanced degenerative disc disease (*). If conservative management fails, discography should be considered. In a negative discography, we need to rule out other causes of back pain and continue conservative management. In a positive discography with pure axial back pain in advanced disc degeneration, the less invasive option of injection to pain generators, such as epidural steroid injection, percutaneous epidural neurolysis, peri-radicular injection, and facet joint blocks, can be considered for temporal relief of symptoms. Clinicians can consider other more definitive palliative management, such as radiofrequency ablation, which can be applied by minimally invasive techniques, such as endoscopic radiofrequency ablation of the sinuvertebral, basivertebral nerve, and disc, or fluoroscopic-guided percutaneous intervertebral disc treatment, such as radiofrequency ablation of the disc. Neural compression without instability or disc collapse can be treated with decompression only for patients without discogenic back pain. While for patients with discogenic back pain and neural compression, decompression surgery with additional endoscopic or other methods of treatment of the sinuvertebral and basivertebral nerve can be done [12]. Total disc replacement is done when there is disc collapse with the intention to preserve motion; fusion is done when there is disc collapse with the intension of rigid fusion. If there is any symptomatic gross instability, fusion surgery should be considered.

When a patient presents with lower back pain, it is important to correlate clinical symptoms with imaging. If symptoms are not concordant with the imaging modality, interventional treatment may not yield the benefits desired. In fact, there is a limited correlation of disability with the degree of disc degeneration in the MRI findings of several studies [49,50]. A trial of conservative management, such as physiotherapy, oral analgesia, and supplements with or without alternative medicine, would help in some patients with DDD. However, the recurrence of lower back pain is common when ignored [51]. The recent developments in imaging and inflammatory markers may help in decision making in the near future. Provocative discography was the gold standard provocative test for degenerative disc disease. However, it is not a routine practice performed in all degenerative disc disease as it is an invasive procedure with inherent risks, such as infection and neural injuries [46]. The patient can consider provocative discography when there are significant symptoms despite being compliant to conservative management. When there is concordant pain with discography, a decision can be made based on (1) early versus late stage of DDD, (2) stability of the spinal column, (3) status of neural compression, and (4) predominant symptoms. A summary of the proposed treatment algorithm for early DDD and late DDD is shown in Figure 3 and Figure 4. The details of these treatment strategies are discussed in the following discussion.

### 9.1. Treatment Options for Relief of Pain in Conservative Therapy

#### 9.1.1. Physical Strengthening and Physiotherapy

A trial of conservative therapy is recommended before considering any invasive procedures. Conservative therapy entails physical exercise focusing on back muscle strengthening, physiotherapy, oral medications, and supplements.

Physical exercise is clinically recommended in several guidelines to help in alleviating pain [52]. Physical exercise helps in IVD cell proliferation in animal model studies, particularly in moderate to high volume low repetition and frequency exercises [53,54]. It has an effect on paraspinal muscle strength and aids in reducing pain and disability [55]. Up to 80% of patients with a prolapsed intervertebral disc respond to conservative therapy in an average of 4 to 6 weeks [56,57].

Kjaer et al. showed that most lumbar disc herniation (65%) does not change in size over a 4- to 8-year period, with 17.5% decreasing in size, 12.5% increasing in size, and 5% showing various changes in disc sizes. Large disc herniation tended to decrease the dural sac area and disc height over time [58]. Hence, the role of conservative therapy is mainly on improving the physical well-being of the patient and provides a platform for adaptation of the body while waiting for the inflammatory phase of disc herniation to subside.

#### 9.1.2. Oral Medications

Generally, paracetamol and non-steroidal anti-inflammatory drugs (NSAIDs), opioids, and muscle relaxants are given to patients who present with symptomatic degenerative disc disease with no contraindications to these drugs. These medications are given on top of back education, reassurance, and self-management options. Early return to activity in patients with acute back pain is encouraged. Chronic lower back pain needs multimodal rehabilitation on top of the treatment given for acute back pain [59]. These medications provide short-term pain relief but have no effect on the progression of disc degeneration [60].

There is poor evidence on glucosamine and chondroitin having any effect on the pain or regeneration of DDD from very few literature reports, with conflicting findings [61].

Omega 3 fatty acids (fish oil) as an anti-inflammatory alternative to NSAIDs for discogenic pain have showed low level evidence in both animal and human studies. It seems to be a safe alternative in view of its minimal side effects. However, it is at best a weak recommendation due to its lack of evidence [62,63].

#### 9.1.3. Pain-Relieving Injection

The goals of symptomatic pain relief by injection are (a) decreasing inflammation around symptomatic nerves, (b) providing temporary anesthesia in the specific target area where the irritated nerve is involved, and (c) adhesiolysis between the neural elements concerned and the degenerated/ prolapsed disc through the hydrostatic pressure effect of introducing a volume of fluid in the region of concern.

First and probably the most important step is for the treating physician to determine the area of pain in IVD diseases. In advanced cases of IVD diseases, there is often multiple regions of IVD diseases in the MRI scan. There are typically multiple levels of disc degeneration with associated spinal canal central, lateral recess, and foraminal stenosis as a result of the decrease in disc height, facet hypertrophy from arthritis, and thickening of the ligamentum flavum. Facet arthritis and DDD can be separate causes of lower back pain, which presents in the same manner Hence, this gives the treating physician a significant diagnostic dilemma in which the level and area is the pain generator. Often the patient clinically presents with a mixed picture of pain, and the pain is aggravated on many axes of movement. Overall, the diverse clinical and radiological presentations of a chronic back pain patient with IVD diseases presents a diagnostic challenge. Nerve root and joint block serves as a good diagnostic tool on top of its therapeutic effects. This section briefly highlights the areas of injections and the effects.

ESI for back pain can be given by several routes. Common routes of injections are interlaminar [64,65], transforaminal [66], and caudal injection [67]. ESI is given in the epidural space of the spinal cord. The area of effect for ESI for disc degeneration is usually wider than the perineural region. Hence, it is less helpful as a diagnostic tool than peri radicular injection. The effect of ESI is not definitive, and the long-term success rate is variable.

The spread of an epidural steroid injection is limited in areas of the spine where adhesions are formed from ruptured disc, previous spinal surgery, and chronic degeneration of disc and flavum, cyst, etc. Adhesion lysis aims to provide hydro dissection on the interphase of dura and the pathological tissues [68]. Lee et al. performed percutaneous adhesiolysis on 86 patients with chronic lower back or leg pain who were not responsive to transforaminal epidural injection, and showed efficacy immediately in 2 weeks and up to 3 months in 60‒70% of improvement in their back and leg pain as well as the overall outcome score [69].

Peri radicular injection is also known as selective nerve root block. It is indicated in patients with symptoms of nerve root irritation. Kanayama et al. from Hokkaido University performed peri radicular injection with good clinical outcomes in 42% in disc herniation with no significant complications. However, the efficacy was limited in cases of herniated disc with spinal stenosis and foraminal disc herniation, which caused pain with mechanical compression. Although it is a large study with 641 patients, obviation of surgery is multifactorial, as natural progression of disc herniation is associated with the majority improving with conservative treatment. Nevertheless, with a low complication rate, it is a viable treatment for selected patients [70].

Overall, epidural corticosteroid injections and peri radicular corticosteroid injection for radiculopathy can lead to immediate improvements in pain and possibly improvements in function, but the effect is non sustained. However, it has no effect on the long-term risk of surgery. Evidence does not show that the effectiveness of the injection has a dose-dependent effect on the amount of corticosteroids, frequency, and variation of the technique. Corticosteroid injections are helpful in radicular pain but are less effective for spinal stenosis, non-radicular back pain, and facet joint pathology [71].

### 9.2. Treatment with Aims of Restoration, Repair, and Regeneration of Intervertebral Disc Diseases: Molecular Therapy

Developments in molecular science have led to an effervescence of growth in the development of various experimental and clinical trials in the manipulation of cells, genes, and various growth factors in an attempt to produce end proteins that can repair and regenerate the degenerated disc, typically in the early stages of disc degeneration, i.e., Pfirrmann grade 1–3 groups of patients, in order to restore the damaged intervertebral disc [23]. The three main mechanisms are (1) cell therapy, which works by exogenous injection of cells to augment and replenish the Extracellular Matrix ECM through stem cell, native disc, and chondrocyte cells maturing into cells with anabolic generation capabilities; (2) growth factor therapy, which works through exogenous protein injections that increase the native chondrocytic cell production by upregulation of anabolic extracellular matrix (ECM) proteins and downregulation of catabolic end products; and (3) gene therapy, which works by the transfer of genetic material, which aids to restore or maintain the ECM. Molecular therapy development is promising, with the allure of potentially curing intervertebral diseases. This is different from other treatment strategies, which aim at relieving pain, altering the nature of the diseased disc segment, or definitive replacement and fusion surgery. However, molecular therapy is still largely at the clinical trial and/or experimental stages of development, and widespread advocacy of molecular science practice in clinical medicine is not prevalent.

#### 9.2.1. Cell Therapy

Intervertebral discs are avascular and aneural; yet, during disc degeneration, we often find blood vascular invasion, calcification tissue, and fibrocartilage-like tissue proliferation [12]. These changes are likely mediated by resident stem cells. Resident stem cells are not from the intervertebral disc, and are derived mostly from bone marrow, adipose tissue, and synovium [72].

The principle of cell therapy is the usage of (a) de novo cells, which are nurtured in the laboratory environment to be introduced to the disease region through cell transplant. These cells mediate paracrine signaling, which stimulates the endemic cells to produce favorable end products to rejuvenate the target degenerated disc; or (b) de novo cells directly participate in extracellular matrix (ECM) production and homeostasis [73]. The cells generally used are (1) notochordal cells from embryonic human nucleus pulposus, which diminished rapidly after birth; (2) chondrocytes, which can be obtained as an autologous mature form or allogeneic juvenile form; or (3) mesenchymal stem cells, which can be obtained from autologous bone marrow and adipose tissue or allogeneic form from embryonic umbilical cells [74,75]. The mechanisms involved are (1) cell harvest, (2) cell expansion where 5-10 million cells introduced into the defect or diseased region expand by growing in the monolayer to encourage proliferation, (3) addition of a scaffold or carrier to the cell, and (4) insertion of the cell and scaffold through a minimally invasive method, for example, through a percutaneous needle under image or visual guidance. The process of a cell going through all these processes yet upholding its regenerating potency despite the relatively harsh conditions of an acidic, osmotic, hypoxic, and hypoglycemic environment with limited hydration and intensified catabolic secretion is challenging [76]. Only cell populations that are able to survive and contribute to ECM production while maintaining anabolic features within IVD can be effective in producing the changes in disc regeneration [77,78,79,80].

While stem cells are settling down biologically, there are constant challenges of mechanical loading and changes in osmotic conditions, which can affect the gene expression according to the direction and frequency of loading [81]. There is a significant effect of the osmolarity of IVD on MSC. An experiment showed an inhibitory response to MSC in an IVD-like environment of osmolarity around 485mOsm [76].

James et al. showed that MSC treatment of an IVD lesion in sheep prevents fatty infiltration and fibrosis of the multifidus muscle, but there was no significant slow to fast muscle fiber transformation. Gene expression of proinflammatory cytokines within the muscle was altered by the MSC treatment of IVD. Increased interleukin-1β expression was prevented in the early treatment group and tumor necrosis factor and transforming growth factor-β1 expression was upregulated at 6 months. Such gene expression may have a role in the pain and inflammatory cycle of the IVD [82].

Advantages of cell therapy are that the cell is injected in the nucleus contained by annulus, preventing cell migration, and it is in an immune-privileged milieu [83].

Nevertheless, despite all the difficulties in many phase 1 and animal trials, several authors used various types of mesenchymal stem cells harvested from red bone marrow, fat tissue, stromal vascular faction, hematopoietic stem cells, intervertebral disc chondrocytes, and human umbilical cord to implant in patients with degenerative disc disease as clinical trials, with variable trends of generally positive results and safety profiles [84,85,86,87,88,89,90,91,92,93]. Although, broad usage on more patients with DDD should be exercised with caution due to a lack of long-term studies and limited sample size, with many studies lacking a control arm. There is a promising outlook on the development of the science in mesenchymal stem cells in DDD.

#### 9.2.2. Growth Factor Therapy

Growth factor therapy involves the injection of biological factors directly into IVD to promote synthesis of the extracellular matrix, delay degeneration, and stop inflammation [94]. Growth factors are peptides that target receptors to cause cellular actions, such as proliferation, differentiation, apoptosis, and synthesis of proteins. The most known growth factor in spine and orthopedic practice is bone morphogenic proteins (BMPs) and members of transforming growth factor beta TGF-β, stimulating osteogenesis and chondrogenesis [95]. The key limitation with growth factor therapy is the short biological half-lives, limiting it to hours or days, which is especially limiting in degenerative disc disease where the local conditions are even more challenging to the sustenance of growth factors and the lack of stability of growth factors. These limitations create a significant element of variability in the response after the injection of growth factor into the host. Thompson et al. were the first to perform growth factor investigation in mature canine intervertebral disc stimulation, with variable responses in the nucleus, transition zone, and annulus [96]. Numerous in vitro and in vivo studies on various growth factors done primarily in laboratory settings have shown promising effects [97,98,99,100,101,102]. Growth factor therapy aims to introduce growth factors that promote anabolic functioning with upregulation of extracellular matrix proteins, such as transforming growth factor (TGF-beta), insulin-like growth factor 1 (IGF-1), epidermal growth factor (EGF), platelet-derived growth factor, and bone morphogenetic proteins (BMPs). Other growth factors that downregulate catabolic activities by reducing inflammatory cytokines, such as interleukin (IL-1, IL-6), tumor necrosis factor-alpha, matrix metalloproteinases (MMPs), nitric oxide, and prostaglandin E2 (PGE2), are also applied to promote intervertebral disc restoration.

While in vivo animal studies show growth factors improving disc structures, the correlation with an effect on pain improvement is not direct [96,97,98,99,100,101,102].

Plate-rich plasma contains a mixture of proteins and growth factors and has been injected in the foot and ankle as well as in the knee joint with variable effects [103,104]. Chen et al. showed PRP promoting NP regeneration with an increase in messenger ribonucleic acids (mRNAs) production to promote chondrogenesis and matrix accumulation [105]. Though PRP is easy to obtain and administer, current evidence is lacking in terms of dosage requirements, method of preparation, and a true understanding of the mechanism of action [106].

#### 9.2.3. Gene Therapy

Friedmann and Roblin first introduced gene therapy, which introduces genes to target cells by two ways: (1) In vivo gene therapy involves using viral or non-viral vectors to transfect candidate genes that can be incorporated into the target cells; and (2) the ex vivo method involves getting the target cells out into a culture medium, altering its target genes, and re-implanting them back into the target organs [107]. Interest is growing in this area of development. The attractiveness of modulating the biological activities of IVD by delivering therapeutic genes has led to significant research in this area. Most of the studies are still based in laboratory settings. The advantage of gene therapy is that unlike growth factor therapy, its effect is potentially long term once successful transfer of the gene to native target cells occurs. The target cells with gene alteration will continue to produce proteins favorable for the maintenance and recovery of the intervertebral disc. However, there are challenges in gene therapy as it involves the active transfer of genetic material, generally using a viral vector, with an inherent risk of virus-related complications (such as viral mutations, systemic viral infection, immune response, etc.) So far, gene therapy applications are limited to medically life-threatening conditions, such as cystic fibrosis and sickle cell disease, rather than degenerative conditions. The use of non-viral vectors is not as reliable in genetic transfection [108,109,110,111]. These are current challenges limiting the amount of research in gene therapy. A well-designed gene therapy clinical trial would have good potential in the development of treatment of IVD diseases.

Overall, molecular therapy is a promising technique, which is minimally invasive, motion preserving, and restorative in treating early symptomatic early phases of degenerative disc disease. More investigations into the effects of molecular therapy are in sync and complementary with the developments of a better diagnosis and other treatment modalities of degenerative disc disease.

## 10. Reconstructive Strategies: Percutaneous Intervertebral Disc Techniques

Percutaneous decompression has several different techniques, with different methods and science behind each of these techniques. Their common aim is separation of the neural elements from irritant pathology, reduction of the size of the disc protrusion in the spinal canal, and reconstruction of the function of the damaged IVD. The examples of percutaneous decompressions are mechanical decompression, thermal decompression, chemical decompression, and biomaterial implantation. Such techniques aim to reconstruct the structure and shape of the intervertebral disc in order to relieve the symptoms of the patients. Ideal candidates who are suitable for these treatment strategies are those who have advanced stages of DDD in the MRI scan that present with significant mechanical back pain with positive discography. Disc prolapse, in these cases, should be small and contained as these indirect decompressive techniques are unable to retrieve intracanal disc herniations.

### 10.1. Mechanical Decompression

Spiral tips, metallic wires, and water- and air-driven suction cutting probes are introduced using CT guidance or fluoroscopic guidance to the pathological area and remove disc material anterior to herniation, and through a reduction of the size of the disc material, indirectly decompress the spinal canal [112,113]. In fact, this is the early form of a minimally invasive procedure prior to the evolution of endoscopic spine surgery with more advanced techniques [114,115]. The effect on disc removal is variable, with reports showing good clinical outcomes. With the evolution of minimally invasive spine surgery and endoscopy [116], there is a shift of a non-visualized indirect reduction of disc material through fluoroscopy to endoscopic treatment of the disc with endoscopic visualization to safely and effectively execute the disc removal and treatment of the painful area [12,114].

### 10.2. Thermal Decompression

Thermal decompression involves applying thermal energy to IVD. Thermal energy can be introduced by different kinds of lasers and radiofrequency probes [113,117,118,119]. The primary goal is to use thermal energy to decrease the inflammatory response, leading to shrinkage of the tissue, reducing its compression on the neural elements and destroying the nociceptive fibers in the periphery of the disc [113,118,119]. Disc-fx is a form of thermal decompression, which uses radiofrequency ablation applied to the high intensity zone and bulging part of the disc. It has been used with good early results [120]. The authors used thermal energy under direct endoscopic visualization to provide sinuvertebral and basivertebral nerve nociceptive pain relief [12]. Thermal percutaneous and endoscopic decompression is widely practiced and the most popular of the percutaneous decompression techniques [9,10].

### 10.3. Chemical Percutaneous Decompression Techniques

Chymopapain is a proteolytic enzyme injected intradiscally for the treatment of herniated lumbar discs. The average success rate is 73% for the elimination of backache and sciatica and was popular in the 1980s [121]. However, 3% suffer serious complications, such as anaphylaxis, which quelled the enthusiasm for the usage of this proteolytic enzyme. Recently, a publication based on the result of 1991–2000 on chymopapain showed good results in chemical decompression, with no significant complications in their series [122]. Currently, it is rare to find physicians offering chymopapain as a treatment option for a prolapsed intervertebral disc as compared to 1980 to the early 1990s when it was very popular. Other chemical percutaneous decompression techniques based on the use of an oxygen–ozone mixture and radiopaque gel like ethanol were recently studied. The injection of this admixture of oxygen and ozone (O2-O3) into the nucleus pulposus reduces intradiscal pressure and has immune-modulating effects. It also causes glycosaminoglycan lysis, and the reaction with fragmented proteoglycans leads to shrinkage and dehydration of the disc [123]. The mixture of gas is also administered to the soft tissue adjacent to inflamed nerve roots on the way out while withdrawing the needle from the disc. There are only a few studies with small numbers on this topic with promising results [123,124,125].

A radiopaque gel like ethanol is another emerging technique of chemical decompression. The effect of ethanol provides dehydration and a reduction in the size of the bulging disc [126]. Its hydrophilic property helps pull the peripheral fluid towards the core of NP, which is injected with ethanol gel. In addition, the entrance of the needle for introducing the gel isbe sealed by precipitation of the gel particles [126,127].

### 10.4. Biomaterial Implantation

To address the limitations of spinal fusion and disc replacement surgery, such as adjacent segment disease, pseudarthrosis, and graft or implant-related complications, research on biomaterial implantation is becoming more popular. There are several advantages in biomaterial implantation: (1) Ease of obtaining and manufacturing the biomaterial as compared to molecular techniques; (2) stability in transport and storage; (3) extensive laboratory testing can be done to find a matching compatible material; (4) potential promotion of endogenous repair of the disc architecture by providing a reconstructed scaffold for native or nurtured mesenchymal stem cells to home in, proliferate, and differentiate into suitable cells for repair and restoration of the IVD; and (5) most of the designs of these scaffolds can be introduced with a clinical needle, which makes the perioperative risk of implantation lower than an invasive surgical procedure. This is a relatively new area of research interest. Biomaterial implants can be done in the annulus fibrosus, nucleus pulposus, or total disc transplantation (annulus fibrosus–nucleus pulposus combination) [128,129].

The implantation of hydrogel-based compounds, such as formulations of polyvinyl alcohol cryogel, mimics the biomechanical properties of soft tissues in the natural lumbar intervertebral disc, with the aim of nucleus pulposus regeneration with an intact annulus fibrosus. The research focus is getting appropriate materials similar to IVD with good integration of the implant into the disc without migration or immune reactions and is sustainable under various biomechanical loads and pressure, leading to restoration of the disc morphology and function. There is only an anecdotal study with promising results [130,131]. Likipanichkul et al. used an injectable fibrin-based hydrogel to repair the inner annulus fibrosus. His technique is able to restore compressive stiffness and resist expulsion of the hydrogel despite repetitive loading in the bovine spine [132]. Borem et al. developed a collagen-based, multi-laminate angle–ply annulus fibrosus repair patch that is able to handle physiologically relevant stresses without failure and improve the functional spinal unit axial kinematics and dynamics. It is also biologically stable in a simulated degenerate environment, supporting annulus fibrosus cell viability in bovine vertebrae testing [133]. There are various forms of collagen, fibrin, polyglycolic acid, hyaluronic acid, demineralized bone matrix gelatin, hydroxyapatite, porcine, and bovine bone matrix gelatin materials tested in animal models with biomechanical loading [128,129].

Gloria et al. developed a customized additive-manufactured poly(ε-caprolactone) scaffold with tailored architectural features as the annulus and a cell-laden collagen-low molecular weight hyaluronic acid-based material as the nucleus. The overall structure mimics the size and shape of the IVD well, with the intention of total IVD replacement. The viscoelastic, mechanical properties, concentration, and stability allowed for the injection of an over 16G clinical needle with matching mechanical properties of the lumbar disc and compatibility with human mesenchymal stem cells [134]. Du et al. used a biomimetic annulus fibrosus-nucleus pulposus composite with circumferentially oriented poly(ε-caprolactone) microfibers seeded with annulus fibrosus cells, with alginate hydrogel-encapsulating nucleus pulposus cells as the core in in vitro testing and in vivo testing with fluorescent imaging for a follow-up clinical study and histological and immunological staining and evaluation in nude mice. This biomimetic scaffold showed positive deposition and extra cellular matrix with good support of native cell colonization and integration while maintaining suitable mechanical properties of IVD [135]. Peng et al. showed that with the usage of an injectable genipin-crossed linked system, the mechanical properties were improved more than the conventional cross-linked decellularized annulus fibrosus hydrogels and yet preserved the capacity of differentiation in mesenchymal stem cells in mechanical evaluation, histological, and MRI imaging in a rat model [136].

Tissue-engineered replacement for the nucleus or the whole IVD has been already considered using several technologies, biomaterials, and cell sources. Most of the studies showed positive results in animal kinematics-based experiments [128,134,135,136,137,138,139].

One of the limitations in current annulus fibrosus repair techniques with biomaterial supplementations is the inability to handle various physiological stresses in the clinical setting of around 1.5 MPa [140,141]. Another challenge is that most biomaterial implantation is radiolucent and also has no specific signal changes in the MRI evaluation. These properties are not ideal for clinicians to evaluate the efficacy of the intervention. Martin et al. tried to address this issue with a radiopaque nanofibrous scaffold that can be evaluated in small and large animal models with good biocompatibility. Such an additional scaffold with other biomaterial with mechanical properties mimicking the disc can be helpful to evaluate the stability of the construct in the clinical setting [142]. However, biomaterial implantation has not been extensively tested in the human setting. Most of the research is laboratory in vitro and animal-based in vivo testing, and more research is required in these promising treatment strategies. There are numerous challenges in crossing over from animal to human testing as there are different sizes in IVD as quadrupeds have a smaller IVD than humans and they have a different shape [143]. Degradation products of the biomaterial should not make the harsh environment of the degenerated disc worse and cause harm to resident disc cells [144]. The criteria of success in animal studies are also different compared to stringent human criteria. Most of the studies in biomaterial implantation focus on the compatibility and biomechanical properties of biomaterials, and their effect on pain management is unknown till further evaluation in human trials is performed. With all these factors taken into consideration, we can expect more research and possibly imminent clinical trials on human subjects prior to widespread implantation of biomaterial to reconstruct diseased IVD.

## 11. Definitive Treatment for Intervertebral Disc Diseases

### 11.1. Surgical Management

Surgical management for IVD diseases has been a constant source of debate among surgeons, pain specialists, and scientific communities. Surgical treatment caters to two groups of patients with different needs. The first group of patients with acute deterioration of neurology and/or cauda equina syndrome has a strong indication for early surgical management. Fortunately, that is usually a small group of patients with IVD diseases presenting in such an acute manner. The more common group consists of patients with chronic lower back pain, with imaging showing a degenerative disc with or without disc herniation. In our experience, patient selection is key to good outcomes in the surgical management of patients with chronic back pain. A motivated patient with concordant clinical symptoms and investigations who failed conservative management should be evaluated by either provocative discogram or undergo diagnostic injection to assess for temporarily relief of symptoms. Fulfilling these stringent criteria would help to select the more ideal candidates for surgical treatment. The Swedish Lumbar Spine Study Group provided evidence of significant clinical improvement in back pain and disability in patients who underwent fusion compared to conservative management with physiotherapy in a well-informed selected group of patients [145]. The same group studied the economic cost of surgery as compared to conservative management. They found that while there is a higher short-term cost in 2 years for the surgery group, the long-term treatment effects with its associated economic benefits suggests surgery is superior to conservative treatment, with twice the number of patients returning to work when fusion was done [146]. Several randomized controlled trials subsequently also showed positive effects of surgery in patients who failed conservative management [147,148].

### 11.2. Clinical and Radiological Factors Predictive of Conservative Failure

The most common indication for surgery is failure to improve with conservative treatment [149]. The challenge is therefore to be able to select patients with factors predictive of failure of conservative therapy. Some of the predictive factors suggested in the literature were anterior-posterior fragment size versus the dura thecal sac size ratio. One can predict that for patients in whom the AP diameters of the disc fragment and thecal sac are equal, the likelihood of the need for surgery is increased. Other criteria, such as cerebral spinal fluid flow blockage and a herniation disc location that is closer to the foramen further from the midline, are also more likely to need surgery. The foramen ratio of normal in comparison to the side of the foramen with disc herniation is also predictive of necessity in surgery. Pfirrmann’s grade higher than grade 2 is generally a positive prognostic factor for surgery [150]. While most of these parameters are based on the MRI scan, the overriding factor for most surgeons to offer surgery in patients is the presence of significant morbidity and disability from symptomatic IVD diseases in these patients.

### 11.3. Surgical Options for IVD Diseases

#### 11.3.1. Disc Herniation

Recommended treatment for disc herniation is generally conservative management but surgery should be considered when there is failure of conservative management or when there is significant neurological deficit. Surgery for disc herniation has the main primary aim of decompression of neural elements with removal of the herniated disc fragment. Fusion for primary disc herniation is uncommon, unless it is a case of recurrent herniation that is recalcitrant to decompression and discectomy surgeries or there is another indication of fusion, such as spinal stenosis with instability, spinal deformity, and adjacent segment disease from a previous spinal fusion etc. with concurrent disc herniation at the same level. Approaches to decompression are similar to that of spinal fusion, but it is usually done by an open or minimally invasive microscopic approach through posterior and paraspinal approaches [151,152,153].

**Figure 5:**
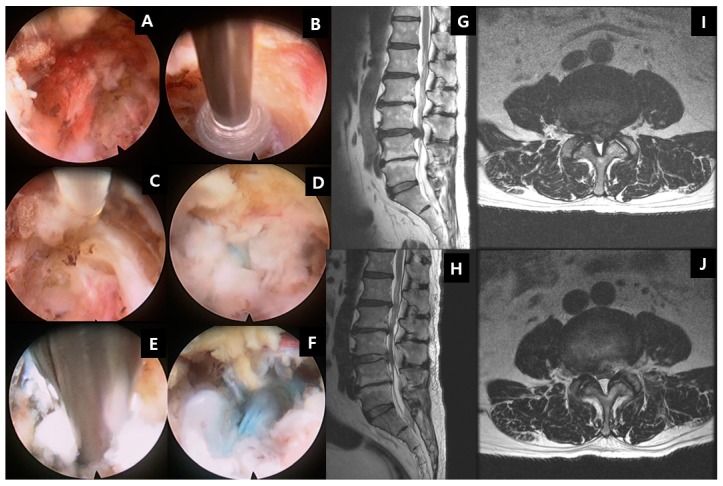
Transforaminal endoscopic lumbar discectomy (TELD). Using a beveled-type working cannula with an 8 mm outer diameter. The endoscope is introduced into the surgical field, with the entire procedure performed using constant saline irrigation. **A** shows the exiting nerve root in view with its surrounding epidural fat tissue. **B** shows the drilling of the superior articular facet under direct endoscopic vision. **C** shows the dissection of the foraminal ligament. **D** showes the exposure of the disc after manipulation of the working channel and endoscope to bring the disc in view. **E** shows the removal of the disc with endoscopic forceps. **F** shows the decompressed disc underneath floating neural elements. The blue stain on the disc was caused by indigo carmine disc injection, which served as a colored marker for ease of identification of disc herniation. **G** shows the herniation of the left L3/4 disc compressing on the neural element in sagittal MRI, which was removed and demonstrated in post-operative MRI of the same level sagittal cut in **H**. **I** shows the herniation of the left L3/4 disc compressing on the neural element in the central, paracentral, and foraminal region axial MRI, which was removed and demonstrated in post-operative axial MRI of the same level axial cut in **J**.

Minimally invasive decompression surgeries can be performed by mini open or tubular retractor with microscopic decompression or the latest technique of endoscopic spinal decompression and discectomy [154,155,156] (Figure 5). With endoscopy, in addition to the posterior and paraspinal transforaminal approach (Figure 5), and a new contralateral approach is available [157,158] [114,159,160]. The long-term outcomes are similar between the open and minimally invasive and endoscopic approach. The soft tissue dissection-wise, endoscopic is least invasive compared to tubular microscopic decompression surgery, which in turn is less invasive than open surgery [161,162].

No matter which approach is used in discectomy, the reoperations rate is significant. Kim et al. showed in a follow up of 1856 patients who underwent surgery for a herniated intervertebral disc a cumulative recurrence rate of up to 16% in 10 years. The probability of reoperation did not differ among open discectomy, laminectomy and discectomy, endoscopic lumbar discectomy, and fusion during the 10-year follow-up period. However, open discectomy was the most commonly used technique by surgeons when performing reoperation [163].

#### 11.3.2. Degenerative Disc Disease with or without Spinal Stenosis

Surgical options for degenerative disc disease are broadly classified according to the goals of surgery, which are decompression, replacement, and fusion.

**Figure 6:**
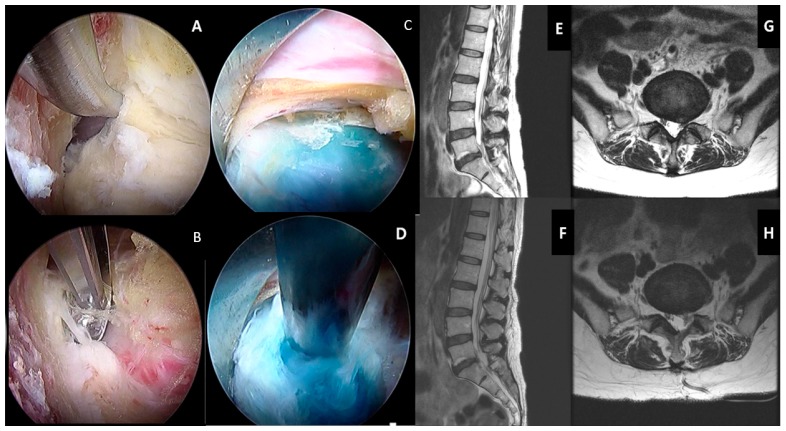
Posterior lumbar endoscopic unilateral laminotomy with bilateral decompression and discectomy over left lumbar five and sacral one. **A** shows the ligamentum flavum was lifted off from the caudal attachment in left L5/S1. **B** shows detachment of the remnant ligamentum flavum from the superior articular process of left side of S1. **C** shows the bulging disc, which is stained with indigo carmine. **D** shows discectomy performed using endoscopic forceps. **E** shows a prolapsed disc with neural element compression in the sagittal view MRI, which is decompressed in the same cut sagittal MRI in **F**. **G** shows a prolapsed disc with paracentral traversing nerve root compression with background thickening of the ligamentum flavum and spinal stenosis in the axial view MRI, which is decompressed with the ligamentum flavum removed and discectomy performed in **H** of the same cut axial MRI.

Spinal decompression is a form of surgery, which has the main goal of removing the bony and soft tissue element to relieve pressure on the neural elements. The areas of neural compression can be central, lateral recess, foraminal, and extraforaminal compression. The indication of decompression surgery is patients who suffer from spinal stenosis and have failed conservative management, and a radiological and clinical evaluation that shows no spinal instability. DDD contributes to neural element compression by a combination of the following mechanisms. As the disc degenerates, the disc is unable to maintain physiological stress and height, hence the disc bulges out into the spinal canal. Its consequent loss of disc height places stress on the facet joint, which leads to facet hypertrophy with bony osteophytes, causing compression to the neural elements. The facet joint may form a facet cyst as a result of the degeneration with synovial joint capsule disruption. As the facet joint subluxed and disc height decreased, the tension on the ligamentum flavum loosened and it buckled inwards and hypertrophied the ligamentum flavum, leading to compression on the neural elements [21]. Decompression surgery involves removing a part or the whole lamina and ligamentum flavum with/without discectomy. Lamina can be removed as a whole (laminectomy), or partial resection of some parts of the lamina to access the underlying ligamentum flavum (laminotomy). After the lamina is removed, exposure and resection of the underlying ligamentum flavum and removal of pathological elements, such as facet cyst, hypertrophied facet joint, and syndesmophyte, and bulging disc, is performed (Figure 6).

Total disc replacement is a procedure that involves the removal of the intervertebral disc and replacing it with an artificial disc, which is typically made up of metal and plastic parts that are bioinert and with mechanical properties suitable for spine biomechanics [164,165]. The joint movement is mostly preserved while aiming to take away the pathological disc, leading to pain relief and an increase in the foraminal height. Total disc replacement is usually done by an anterior approach [165]. The indication of disc replacement is similar to that of degenerative disc disease with stenosis. It can also be performed for degenerative disc disease without spinal stenosis only on selected cases who failed conservative management and suffer from severe morbidity and disability. Overall, a recent meta-analysis showed significant improvement of ODI, satisfaction, and lower reoperation rate than fusion in a 5-year follow up [166]. This study corresponds with the findings of an earlier study by Nie et al. [167]. Total disc replacement is also doing better than anterior lumbar interbody fusion in short-term efficacy and a safety review [168]. It is also of note that lumbar disc replacement goes through the abdomen or retroperitoneal structures; there is less need for muscular dissection and hence less perioperative pain. When well executed with no injuries to iliac vessels, there is less muscular bleeding as compared to the posterior approach of lumbar fusion.

**Figure 7:**
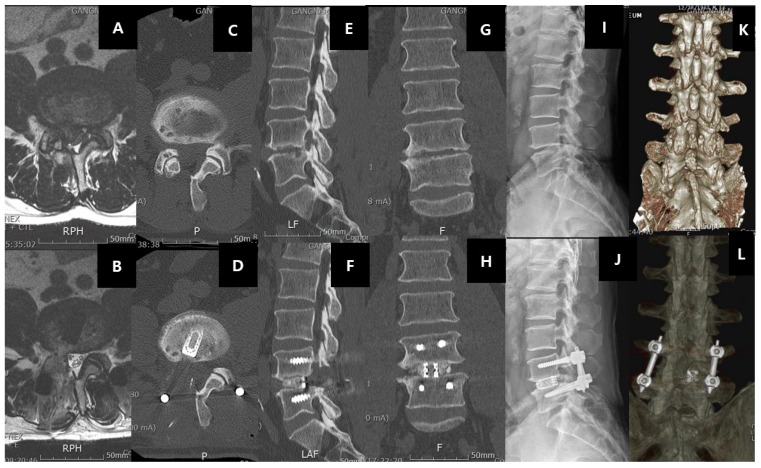
Right-sided L4/5 recurrent disc herniation after previous open laminotomy and discectomy 2 years ago, the patient underwent uniportal endoscopic transforaminal lumbar interbody fusion of right L4/5(ETLIF). **A** shows the recurrence of disc herniation with facet arthritis in the MRI axial cut of L4/5. **B** shows the same axial cut with left transforaminal lumbar interbody fusion showing resection of the facet joint and cage in an optimal position. **C** and **D** show the pre and post-operative status of the right facet. Note the previous right L4 laminotomy in **C**, the facet was resected, and the cage introduced in an optimal position as shown in **D**. **E** and **F** show the sagittal view of the pre and pos-operative status. Note the increase in the foraminal height and intervertebral height as a result of the right L4/5 ETLIF. **G** and **H** show the increase in the coronal disc height pre and post-operatively in right L4/5 ETLIF. **I** and **J** show the pre and post-operative standing neutral XR with the L4/5 interbody cage and standard posterior inserted pedicle screws in L4 and L5. **K** and **L** show the 3D reconstruction of pre and post-operative right L4/5 ETLIF.

Spinal fusion surgery is a procedure to join two or more vertebra into one single structure (Figure 7). Spinal fusion typically involves (a) the removal of the joint cartilaginous substance, (b) followed by insertion of a material that has a combination of osteoinductive, osteoconductive, and osteogenic properties. The common substances used are autograft from bone harvested from the patient, allograft from bone banks, bone morphogenic proteins, and other calcium-based substances [169,170,171]. (c) Stabilization of the construct with instrumentation. Successful fusion ensures no movement between the two vertebrae and hence prevents further pathological enlargement of the facet joint, disc bulge, and buckling of the ligamentum flavum, which compress the neural elements. Placement of an interbody cage is a common method of fusion in spine surgery, which can increase the foraminal height by raising the space between the vertebrae [172,173]. The main indication for spinal fusion is similar to that of spinal decompression, with an additional element of instability either as a complication of the disease process or as an anticipated result of surgery (for example, excess bone removal in a revision decompression, which will cause instability). It can also be performed for degenerative disc disease without spinal stenosis. However, it should be done with stringent indication of selected cases who have failed most if not all forms of conservative management.

### 11.4. Recent Development of Endoscopic Spine Surgery and Its Role in Degenerative Disc Diseases

**Figure 8:**
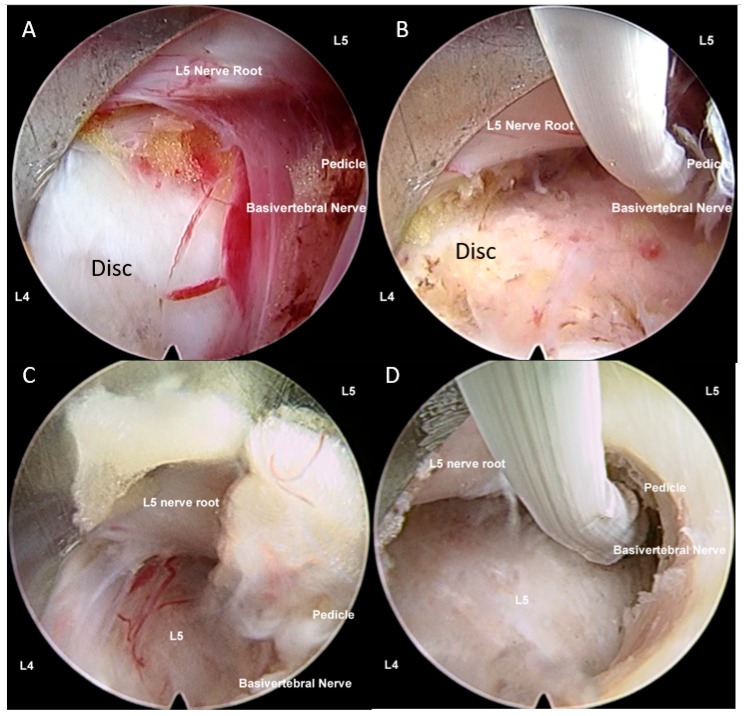
**A:** Uniportal interlaminar endoscopic approach to the disc showing the relationship of the lumbar five (L5) traversing nerve root, which is retracted away by the working channel, exposing the disc of L4/5, the basivertebral nerve is located above the pedicle of left L5, and there is grade 3 neovascularization and inflammatory granulation tissue with adhesion around the basivertebral nerve region. In most circumstances, the basivertebral nerve is too fine to be seen by endoscopic vision. **B**: The same region in the same patient after radio frequency was applied to shrink the degenerative disc, and to ablate the pathological neovascularization with underlying inflammatory tissues and the basivertebral nerve. The typical response is twitching of the buttock when the correct location of the basivertebral nerve is ablated. **C:** Another patient with similar steps in retraction of the traversing nerve root and exposing neovascularized tissue and the location of the basivertebral nerve. **D**: Radiofrequency ablation applied to neovascularized tissue, disc, and basivertebral nerve (figure reproduced with permission from Kim et al. [12]).

With the advancement in minimally invasive techniques, endoscopic spine surgery has recently become popular [114]. Endoscopy also allows spine surgeons to navigate to areas that were not accessible previously, such as the contralateral approach [157]. Having a magnified endoscopic view of the intervertebral disc and dura, one can safely decompress the neural elements of adhesion from DDD, and provide targeted radiofrequency to specific pain-generating nerves, such as sinuverterbal and basivertebral, to cut off the painful stimuli from the disc [12] (Figure 8).

### 11.5. Summary of Surgical Techniques

There are various terms and nomenclature of the various spine procedures can be confusing to young surgeons and scientists involved in spine-related research. In summary, all three types of surgeries (decompression, fusion, and disc replacement) are sub-classified according to (a) the degree of exposure, such as open or minimally invasive; (b) the direction of the approach to the spine, such as the posterior, posterolateral, transforaminal, direct lateral, oblique lateral, and anterior approach [174,175,176,177,178]; (c) in fusion surgery, the type of fusion, such as interbody and posterolateral; (d) in the decompression surgery, the amount of bone removed, such as laminectomy, laminotomy, and foraminotomy; and (e) the instrumentation method and approach required, such as percutaneous pedicle screw fixation, open posterior pedicle screw fixation, and cortical bone trajectory screws. Hence, a typical description of an open approach from the posterior direction with removal of the full lamina and spinous process of lumbar five lamina for a lumbar five–sacral one spinal stenosis is termed posterior open laminectomy of lumbar five vertebra. While the minimally invasive uniportal endoscopic transforaminal approach to fuse the lumbar five sacral one disc is termed posterior uniportal endoscopic transforaminal lumbar interbody fusion (Figure 7). A lumbar five sacral one interbody fusion if done from the posterior approach with standard pedicle screw insertion from the posterolateral direction is called open posterior lumbar five sacral one lumbar interbody fusion. While the same fusion procedure using cortical bone trajectory screws is termed open posterior lumbar interbody fusion with cortical bone trajectory fixation. There are many acronyms, such as ALIF (anterior lumbar interbody fusion), OLIF (oblique lumbar interbody fusion), XLIF (extreme lateral lumbar interbody fusion), TLIF (transforaminal lumbar interbody fusion), PLIF (posterior lumbar interbody fusion), and MidLIF (midline lumbar interbody fusion, which essentially means posterior lumbar interbody fusion with cortical bone trajectory screws fixation), etc.

Most of the comparison studies showed conflicting data on the comparison of decompression, disc replacement, and fusion [179,180,181,182]. There are also controversies over the comparison of full endoscopic, other minimally invasive (mini-open, tubular, etc.), and open surgery [183,184,185,186].

Surgical options of intervertebral disc diseases are controversial, with mid to low quality evidence supporting each of the techniques of decompression and discectomy, fusion, and disc replacement. It is confounded by the surgical experience and technical expertise of the surgeons as well as the comorbidities of the patients. Surgical procedures have inherent risks and complications, hence they should be offered after conservative measures have failed to improve the patient’s quality of life. Overall, the objective of all types of spine surgery in intervertebral disc diseases is to provide a definitive treatment for the patient to maintain long-term pain relief, reduce the rate of complications and reoperations, and provide good patient satisfaction.

## 12. Limitations

The recommendations and proposed treatment strategies are based on the authors’ opinion based on a broad search of the current literature. In an ideal situation, a meta-analysis of each arm of diagnosis and management of IVD diseases would provide a stronger management recommendation; however, that is beyond the scope of this review.

## 13. Conclusions

With an aging population, intervertebral disc disease is becoming more prevalent in society and contributes significantly to the years to life disability. It is confounded by the biopsychosocial factors surrounding patients who present with symptomatic back pain from an intervertebral disc. Precise diagnosis involves concordant clinical and radiological findings. Biomarkers and specialized scans to provide more insight into intervertebral disc diseases are under investigation with a promising outlook. Confirmatory tests, such as a provocative discogram, can be done, but it is not very specific. Conservative management is preferred to surgical management in the initial phase of treatment. Injection of local anesthesia and steroids is a good diagnostic and short-term therapeutic pain relief option. Scientists, physiotherapists, pain specialists, and surgeons all have a role to play in the treatment of intervertebral disc diseases. Recent advancements in restorative and reconstructive treatment strategies in molecular science in cell, gene, and growth factor therapy and percutaneous interverterbral disc techniques are developing in the hope of repair and regeneration of the disc have some challenges but have also shown promising results. Surgery is the last option, with the choice of decompression, fusion, and disc replacement a subject of debate among surgeons, with different backgrounds of patients and surgical expertise further diversifying the choice among the options. A recent development of endoscopic spine surgery provides new insights in the spinal surgery of disc and pain management strategies. It is a truly exciting time of scientific development in intervertebral disc disease as no single therapy has provided enough answers to address this potentially disabling and common condition in mankind.

## Abbreviations

IVD	Intervertebral Disc
DDD	Degenerative Disc Disease
ODI	Oswestry Disability Index
VAS	Visual Analogue Scale
AF	Annulus Fibrosus
NP	Nucleus Pulposus
PLL	Posterior Longitudinal Ligament
TELD	Transforaminal Endoscopic Lumbar Discectomy
IELD	Interlaminar Endoscopic Lumbar Discectomy
SVN	Sinuvertebral Nerve
BVN	Basivertebral Nerve
